# Major Vault Protein/Lung Resistance-Related Protein: A Novel Biomarker for Inflammation and Acute Infections

**DOI:** 10.3390/microorganisms12091762

**Published:** 2024-08-25

**Authors:** John G. Routsias, Dionysia Marinou, Maria Mavrouli, Athanasios Tsakris, Vassiliki C. Pitiriga

**Affiliations:** Department of Microbiology, Medical School, National and Kapodistrian University of Athens, 11527 Athens, Greece

**Keywords:** major vault protein, inflammation, biomarker, infection, vaults, MVP

## Abstract

Introduction: Vault particles are large cytoplasmic ribonucleoprotein particles that participate in inflammation. The aim of this study was to assess the diagnostic and prognostic value of major vault protein (MVP) in patients with inflammation, in order to determine whether MVP could be used as a biomarker for infection or inflammation. We also aimed to compare the diagnostic impact of MVP compared to other conventional measurements, such as CRP or white blood cell (WBC) counts. Methods: CRP and MVP levels were measured in 111 sera samples from 85 patients with inflammation admitted to a tertiary-care hospital and 26 healthy individuals during an 18-month period (2019–2020), using nephelometry and a custom MVP sandwich ELISA assay, respectively. In addition, WBC counts were measured using a commercial assay. Results: MVP levels were found to be elevated in patients with inflammation compared to healthy individuals (*p* < 0.0001). Moreover, MVP levels were higher in patients with inflammation due to an infectious etiology compared to those with non-infectious etiology (*p* = 0.0006). MVP levels significantly decreased during the first four days of infection in response to antibiotic treatment, while CRP levels showed a less-sensitive decline. An ROC curve analysis demonstrated that MVP and CRP have similarly high diagnostic accuracy, with AUCs of 0.955 and 0.995, respectively, followed by WBCs with an AUC of 0.805. Conclusions: The ROC curves demonstrated that MVP has the potential to serve as a diagnostic biomarker for inflammation and infection. Additionally, MVP levels may reflect the efficacy of antibiotic treatment.

## 1. Introduction

In the cytoplasm of eukaryotic cells, vaults comprise the largest ribonucleoprotein particles [1]. Although they belong to the lesser-known cellular molecules, they are highly conserved particles that are present in a wide variety of eukaryotic organisms. Their number range from 10^4^ to 10^7^ particles per cell [2]. Vaults were described for the first time in 1986 [3]. They are composed of three proteins: major vault protein (MVP) (96 kDa); vault poly (ADP-ribose) polymerase (VPARP) (193 kDa); telomerase-associated protein 1 (TEP1) (290 kDa); and small untranslated RNA (vRNA) [4,5,6,7]. MVP accounts for more than 70% of their mass. TEP1 is a telomerase-associated protein that is located at the end of vault caps, serving mainly for vRNA binding and stabilization of vault particles [5,8,9,10]. vRNA comprises a small portion (about 5%) of the mass of vaults. In humans, three different vRNAs (HVG1–HVG3) with a significant sequence conservation have been identified [11]. Recently, a fourth vRNA gene (HVG4) has also been identified [12].

The role of vault particles has not been clearly identified yet. Recent studies have indicated that vault particles may play a role in the inflammation process. It has been demonstrated that MVP suppresses the TLR/IL-1R-associated IKK-NF-κB signal pathway [13]. MVP (or probably the whole vault complex) interacts with TRAF6 and inhibits the formation of the TRAF6-IRAK complex and TRAF6 polyubiquitination. Given that the recruitment of TRAF6 to IRAK promotes its oligomerization and the activation of NF-κB via IKK-NF-κB cascades [13], MVP has a pivotal role in the inhibition of this pathway that triggers the onset of inflammation. MVP can also affect the TNF-α pathway. Previous reports suggested that TRAF6 negatively regulates TNF-α induced NF-κB activation upstream of IKK [14].

Several researchers have also suggested a role for vaults in cancer cells’ ability to resist chemotherapy [15,16]. It is believed that vRNAs can interact with chemotherapeutic compounds and facilitate their entrapment within the vault structure [17]. Moreover, it has been reported that vaults and their components are important regulators of intracellular signaling, cell survival, and DNA repair processes [18,19]. Extracellular MVP has been found in patients with rheumatoid arthritis [20] and in extracellular vesicles secreted by senescent cells [21]. It is unclear if the MVP is released from damaged cells in rheumatoid arthritis, but in the case of senescent cells, they are secreted within extracellular vesicles [21]. Inflammation is a component of natural immunity, which aims at safeguarding homeostasis when it is perturbed by several causes, like infection, tissue damage, or cancer [22,23]. Initially, receptors of the natural immunity system are activated and the production of inflammatory mediators, like the Tumor Necrosis Factor (TNF), interleukin-1 (IL-1), interleukin-6 (IL-6), and chemokines, is induced [24]. These responses result in the secretion of acute-phase positive proteins (e.g., C-reactive protein), mainly from hepatic cells, the production of prostaglandins, which are mainly responsible for the appearance of symptoms like fever, and leukocytosis, through the production/transfer/maturation of leukocytes from the bone marrow.

Building on existing research data that indicate a significant association between MPV and the inflammatory process, the aim of this study was to evaluate the potential of MPV as a marker of inflammation. Given the critical role that accurate and early identification of inflammatory states plays in clinical practice, identifying reliable biomarkers is essential. To our knowledge, this is the first time MPV has been investigated and described as a marker of inflammation, potentially providing a new tool for clinicians in diagnosing and managing inflammatory diseases.

## 2. Materials and Methods

### 2.1. Study Population

Serum samples from patients with inflammation were recruited from the patient population of hospitalized patients in Metropolitan Hospital, a tertiary-care center in Athens, Greece, over an 18-month period (2019 to 2020). The primary selection criterion was the existence of at least one sample of markedly elevated CRP levels (more than 10 times the normal upper limit of 0.5 mg/dL). All principal medical and surgical subspecialties, except transplant and obstetrics, are offered in Metropolitan Hospital and thus no key adult population was excluded. The patients with infections included urine infections, trauma infections, pulmonary infections, pyelonephritis, and gastroenteritis. The patients with inflammation of non-infectious etiology included cancer, surgical inflammation, pleural effusion, respiratory distress syndrome, pancreatitis, and Hodgkin’s lymphoma. Whole blood from peripheral veins of patients and healthy individuals was collected and centrifuged after informed consent was obtained. Serum was separated, aliquoted, and stored at −80 °C until use.

### 2.2. Laboratory Measurements

MVP quantification was performed using a custom MVP sandwich ELISA assay (Novateinbio, Hölzel Diagnostika GmbH, Köln, Germany). An ELISA microtiter plate was pre-coated with monoclonal antibody specific for human MVP. Serum samples were added in a dilution of 1:3 (50 μL/well), and after washing 100 μL of biotin-labeled antibody 100 μL/well was added. After incubation of 1 h at 37 °C, the plates were washed again and of 100 μL of horseradish peroxidase conjugated-antibody (HRP) was added. Incubation of the plate for 1 h at 37 °C followed. The plate was then washed, and chromogen solutions were added and incubated at 37 °C. Finally, stop solution was added, and the optical density (OD) was read at 450 nm.

Concentration of IL-6, human beta-defensin 1 (hBD-1), and human beta-defensin 2 (hBD-2) were measured in serum by commercial enzyme-linked immunosorbent assays (ELISA) (Peprotech EC Ltd., London, UK). The concentration of C-reactive protein (CRP) was measured using a cobas c 503 analytical unit for clinical chemistry (Roche Diagnostics International Ltd., Rotkreuz, Switzerland). In addition, white blood cells (WBCs), neutrophils (Neu), hematocrit (HCT), and platelets (PLT) were measured using the DxH 900 Hematology Analyzer (Beckman Coulter, Inc., Brea, CA, USA).

### 2.3. Statistical Analysis

Statistical analyses were performed using GraphPad Prism version 4.0 (GraphPad Software Inc., La Jolla, CA, USA). Pearson’s test was used for correlation analysis. The significance level was set at 0.05. Comparisons of variables were performed with a chi-square test. Student’s *t*-test method was used to compare the mean levels of quantitative variables that were involved in the inflammation process. Receiver operating characteristics (ROC) analysis was performed to evaluate the area under the curve (AUC) and consequently the diagnostic value of MVP in regard to inflammation.

## 3. Results

### 3.1. General Features of the Examined Cohort

A total of 111 serum samples were included in this study, from which 26 samples were healthy individuals (control group) and 85 samples were patients with inflammation. Specifically, 36 patients had inflammation of infectious etiology, 25 had cancer, 3 had both infection and cancer, and 27 suffered from other inflammatory diseases (Table 1).

### 3.2. Patients with Inflammation Exhibit Higher Levels of MVP as Compared to Healthy Individuals

**Figure 1:**
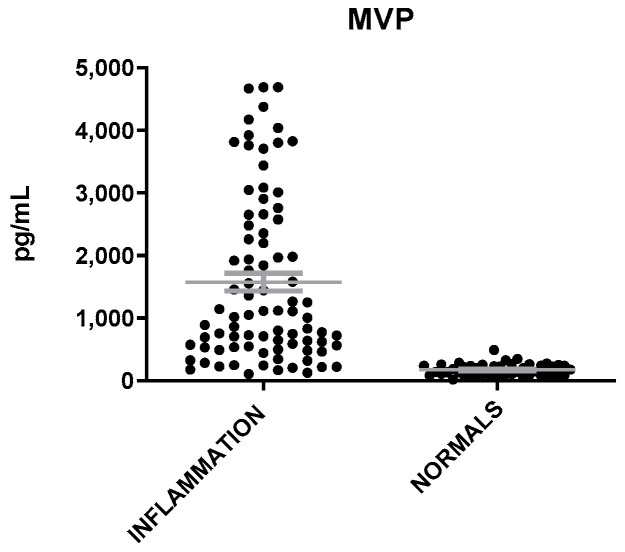
MVP levels in patients with inflammation and healthy individuals. The gray lines represent mean with SEM (Standard Error of the Mean).

**Figure 2:**
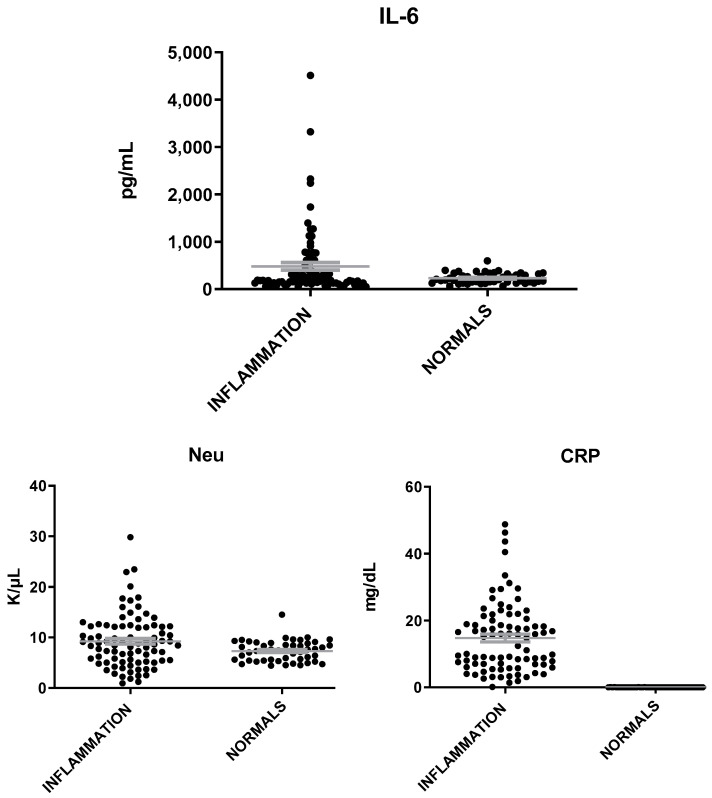
IL-6, Neu, and CRP levels in patients with inflammation and healthy individuals. The gray lines represent mean with SEM (Standard Error of the Mean).

MVP levels were found elevated in patients with inflammation as compared to healthy individuals (t = 7.12; *p* < 0.0001, Figure 1). IL-6 and neutrophils (Neu) were also found to be elevated in patients with inflammation but at a less significant level than that of MVP (t = 2.4, *p* = 0.02) (Figure 2). The mean levels of MVP were 1575 ± 145 pg/mL in patients with inflammation and 172 ± 14 pg/mL in normal subjects. In addition, a significant association was found between MVP positivity and inflammation (x^2^ = 79.81; *p* < 0.00001).

### 3.3. Patients with Infection Exhibit Higher Levels of MVP as Compared to Patients with Inflammation of Non-Infectious Etiology

**Figure 3:**
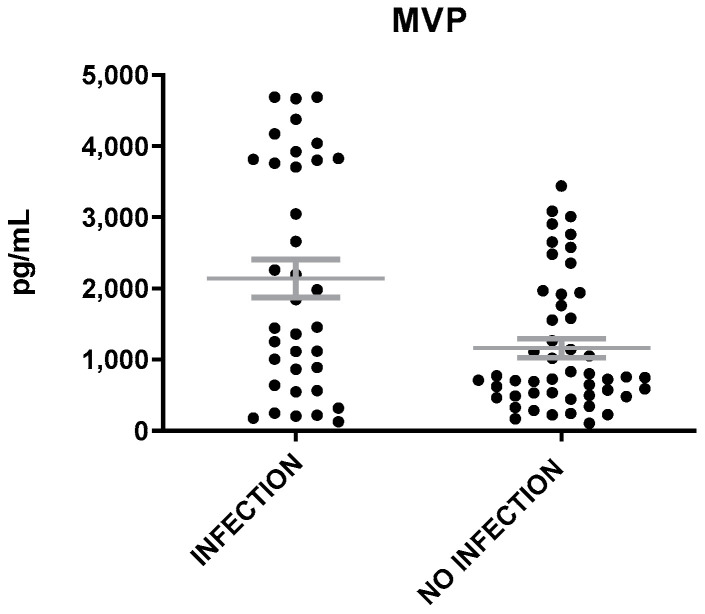
MVP levels in patients with inflammation of infectious and non-infectious etiology. The gray lines represent mean with SEM (Standard Error of the Mean).

**Figure 4:**
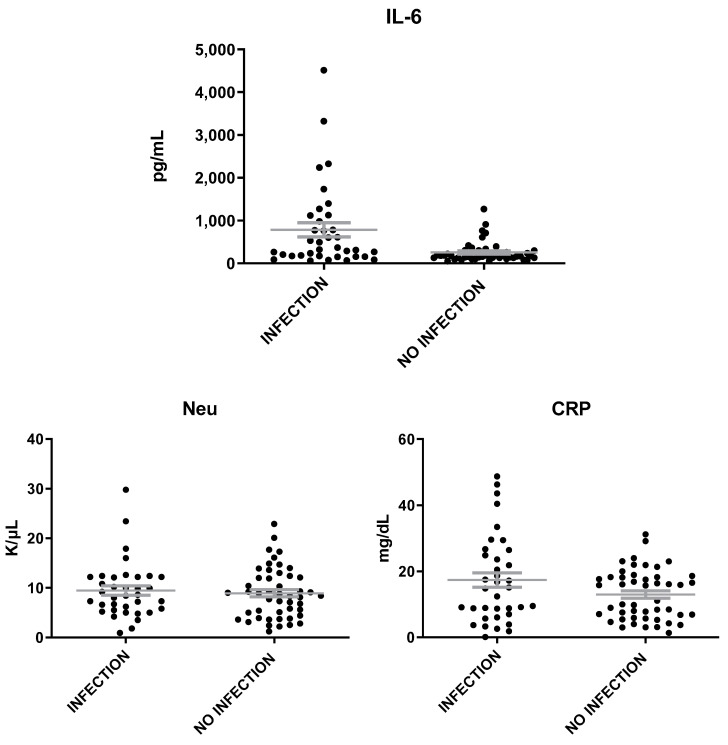
IL-6, Neu, and CRP levels in patients with inflammation of infectious and non-infectious etiology. The gray lines represent mean with SEM (Standard Error of the Mean).

The levels of MVP were heightened in patients with inflammation of infectious etiology as compared to those with inflammation of non-infectious etiology (t = 3.6, *p* = 0.0006, Figure 3). The levels of IL-6 were found also elevated in patients with infection than in patients without infection (t = 3.5, *p* = 0.0007), while the levels of Neu and CRP were similar in patients with inflammation of either infectious or non-infectious etiology (Figure 4). A correlation was found between MVP positivity and inflammation of infectious etiology (x^2^ = 16.14; *p* = 0.00006). The mean levels of MVP in patients with infection (2139 ± 265 pg/mL) were about two-fold increased as compared to patients with no infection (1160 ± 132 pg/mL).

### 3.4. High MVP Levels Are Not Exclusively Associated with Cancer

**Figure 5:**
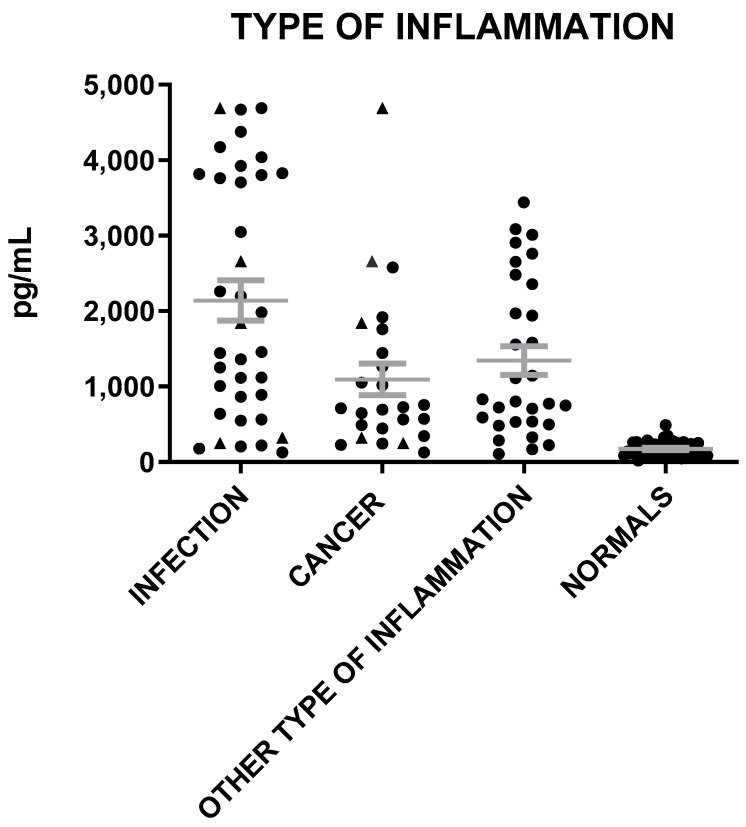
MVP levels in patients with inflammation of infection, cancer, and other types of inflammation and in healthy individuals (triangle represents patients both with infection and cancer). The gray lines represent mean with SEM (Standard Error of the Mean).

**Figure 6:**
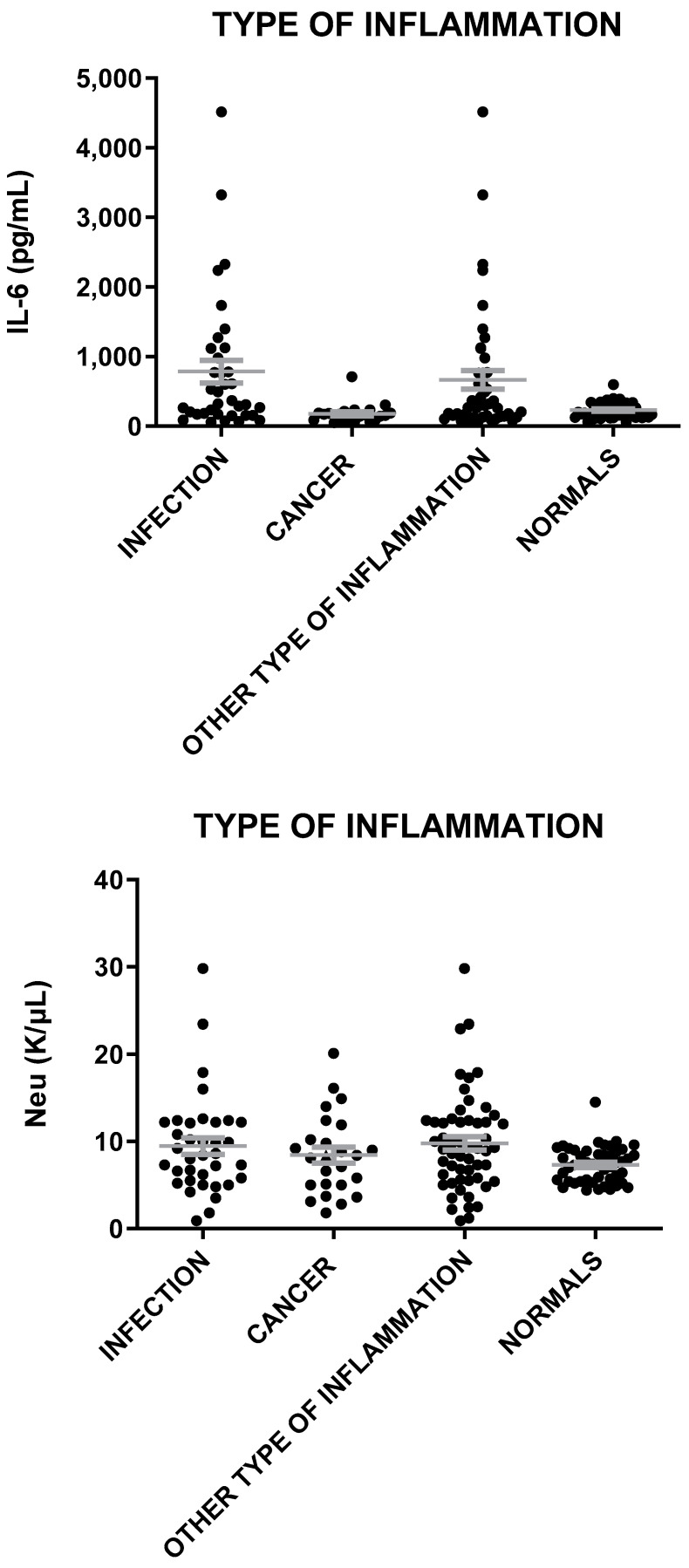

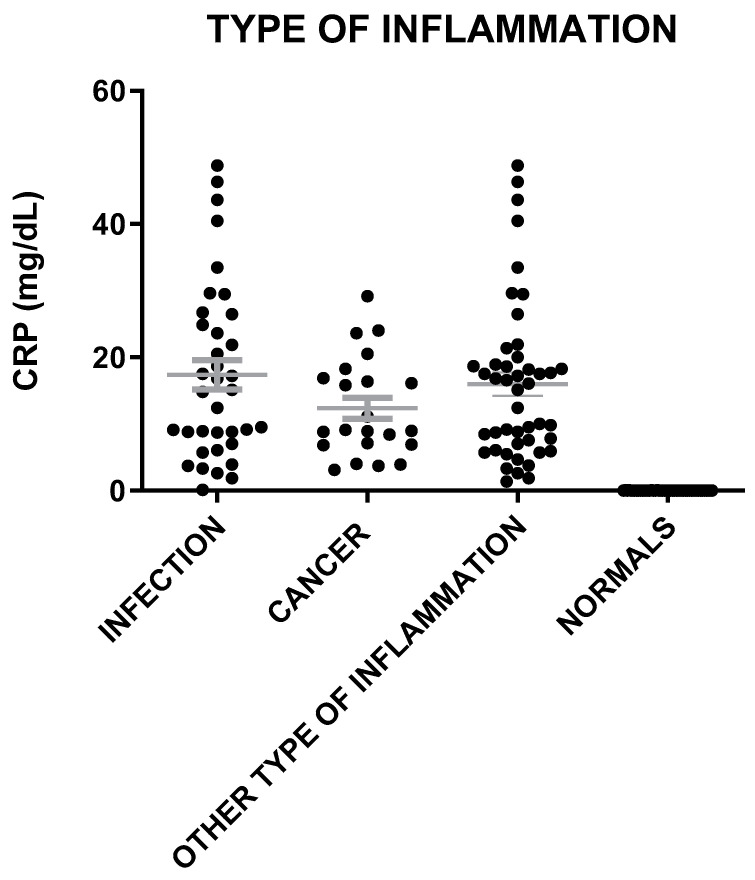
IL-6, Neu, and CRP levels in patients with inflammation of infection, cancer, and other types of inflammation and in healthy individuals. The gray lines represent mean with SEM (Standard Error of the Mean).

The mean levels of MVP in sera were elevated in all cases of inflammation (infection, cancer, other type) as compared to healthy individuals (t = 8.4, *p* < 0.0001; t = 6.0, *p* < 0.0001; t = 7.6, *p* < 0.0001) (Figure 5). MVP levels were lower in patients with cancer as compared to patients with infection (t = 2.89, *p* = 0.006), and they were also slightly lower in patients with cancer than in sera with inflammation of other etiologies (t = 2.24, *p* = 0.028) (Figure 5). Similarly, Il-6 levels were lower in patients with cancer as compared to patients with infection (t = 2.81, *p* = 0.007), while Neu and CRP levels were similar to patients with cancer and the normal controls (Figure 6).

### 3.5. Correlations between MVP and Other Markers/Proteins of Inflammation

**Figure 7:**
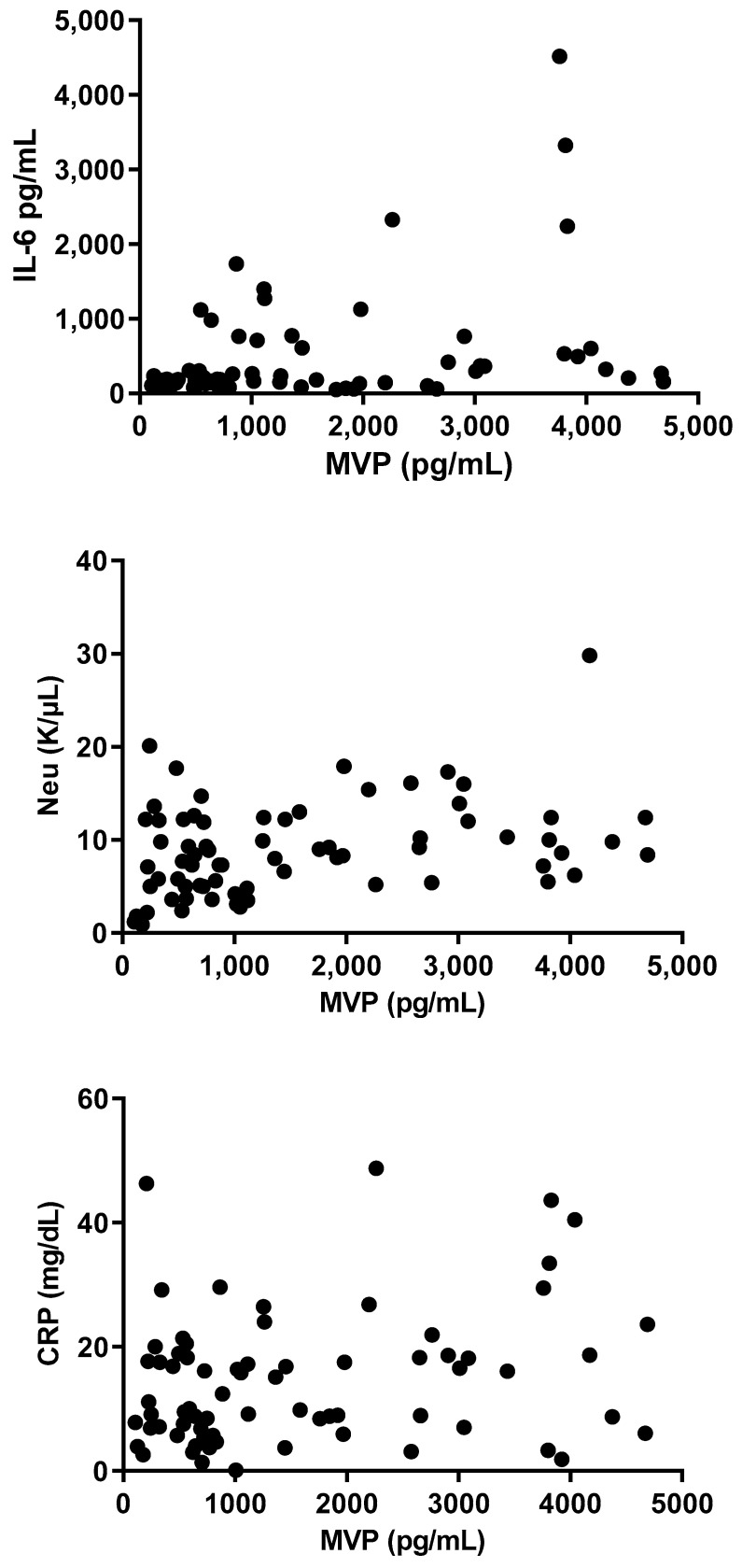
Correlation between levels of MVP and IL-6, Neu, and CRP levels in serum.

**Figure 8:**
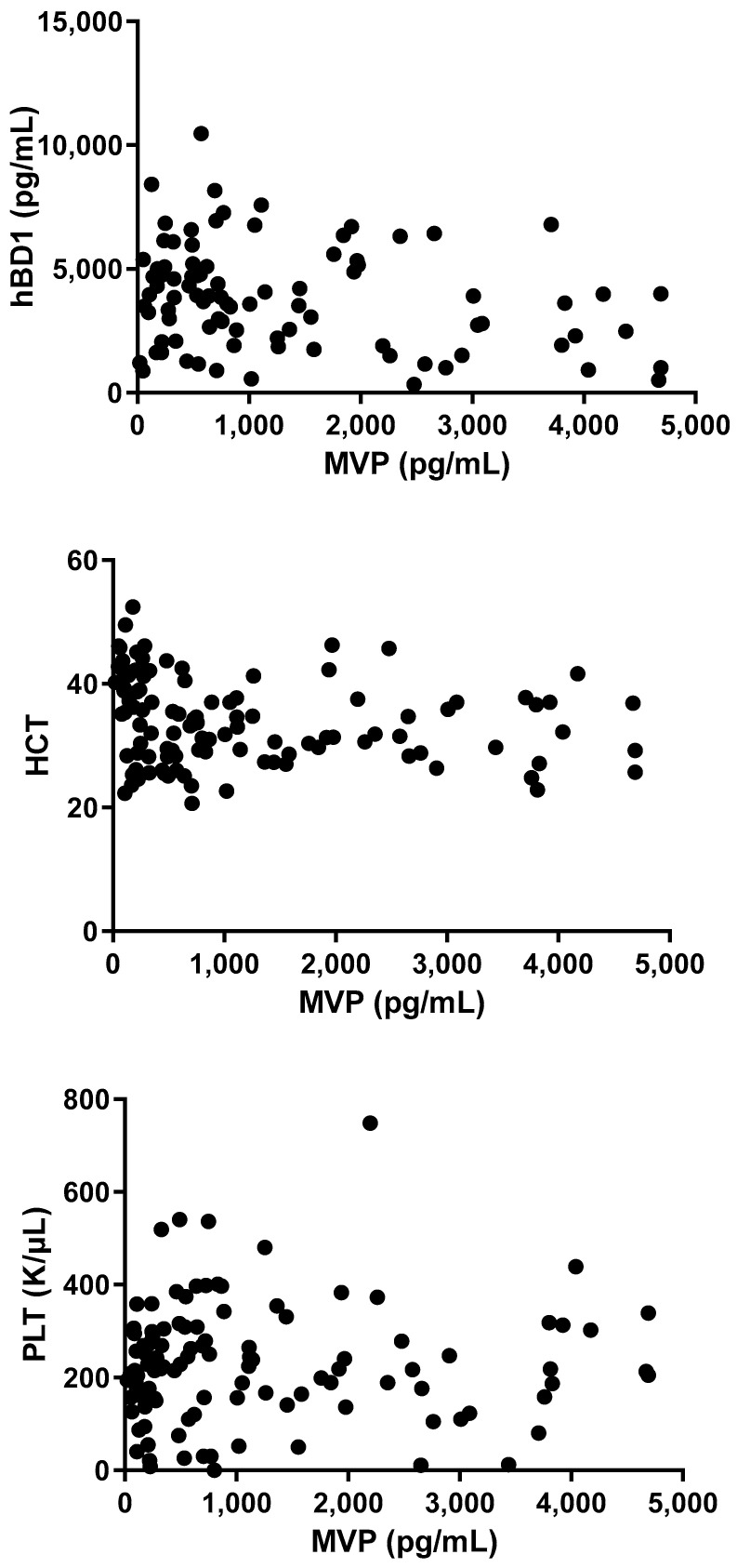
Correlation between levels of MVP and hBD1, HCT, and PLT levels in serum.

A highly significant correlation was found between MVP levels and Neu, CRP, and IL-6 (r = 0.48; *p* < 0.0001, r = 0.42; *p* < 0.0001, r = 0.36; *p* = 0.0004, respectively; Figure 7), whereas a less-significant correlation was demonstrated between MVP levels hBD-1 (r = −0.25; *p* = 0.0170). Finally, no significant correlation was found between MVP and HCT or PLT (r = −0.05; *p* = 0.61, r = 0.036; *p* = 0.71, respectively) (Figure 8).

A multivariate analysis revealed a significant correlation between infection and high levels of MVP (r = 0.12, *p* < 0.0001, cutoff = 395), IL-6 (r = 0.23, *p* < 0.0001, cutoff = 395), Neu (r = 0.14, *p* < 0.0001, cutoff = 9.7 k), and CRP (r = 0.18, *p* < 0.001, cutoff = 0.9), whereas MVP was not significantly associated with hBD-1 (r = 0.023, *p* = 0.086, cutoff = 6234) or hBD-2 (r = 0.028, *p* = 0.058, cutoff = 209).

### 3.6. Diagnostic Accuracy of MVP, CRP, and WBCs for Inflammation

**Figure 9:**
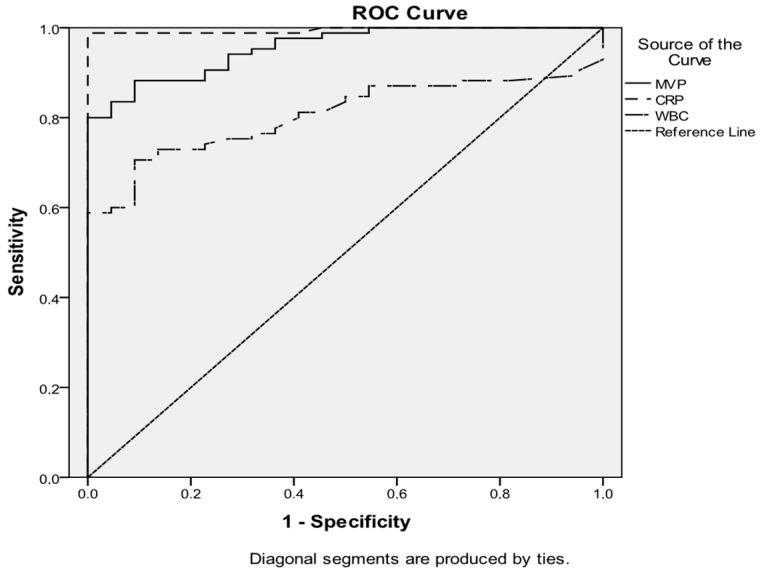
The ROC curve analysis shows the results of MVP, CRP, and WBC predictions of inflammation. The AUC was 0.955 (95% CI: 0.920–0.991), 0.995 (95% CI: 0.984–1.00), and 0.805 (95% CI: 0.725–0.885) respectively.

An ROC analysis was performed to identify the predictive value of serological MVP, CRP, and WBCs regarding inflammation (Figure 9). Based on the ROC curve, MVP and CRP present a similar high diagnostic accuracy with an AUC of 0.955 (95% CI: 0.920–0.991) and 0.995 (95% CI: 0.984–1.00), respectively, followed by WBCs with an AUC of 0.805 (95% CI: 0.725–0.885).

### 3.7. MVP Levels during Inflammation

**Figure 10:**
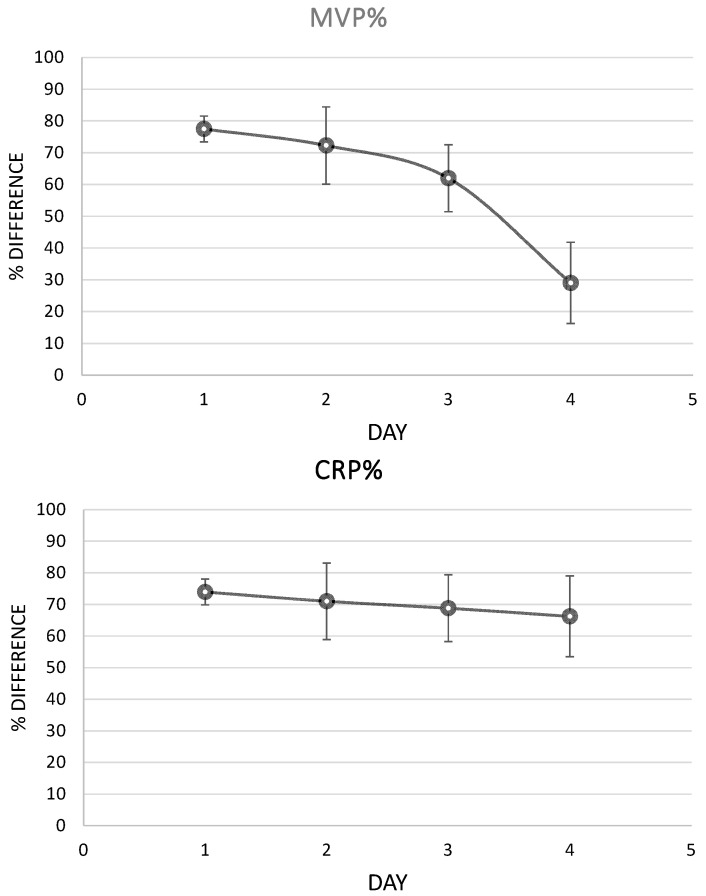
MVP and CRP levels during inflammation. The % difference represents the difference in the marker’s levels regarding its initial levels (the marker’s levels at the first sample that was collected).

Analysis of the serial samples of the patients revealed that MVP had a remarkable fall during the first four days of infection, as a response to the antibiotic treatment, while CRP levels had a lower, insensitive fall (Figure 10).

## 4. Discussion

Recent studies have suggested that vault particles may play a role in the inflammatory process. Therefore, this study investigated serum levels of MVP in patients with inflammation, particularly those with inflammation of infectious or cancerous origin. The findings indicated that MVP levels are elevated in inflammatory diseases, suggesting that MVP could serve as a novel biomarker for inflammation.

Many studies suggest that vaults may be related to inflammatory processes. MVP was found to be enhanced during infection with viruses, including the hepatitis C virus (HCV), vesicular stomatitis virus (VSV), influenza A virus (IAV), enterovirus 71 (EV71), and HBV [25,26]. Similarly, vRNAs are upregulated in Epstein–Barr virus (EBV)-infected human B cells [27]. According to a recent study, *Listeria monocytogenes* masks its surface with human MVP, in order to escape intracellular recognition and avoid autophagy [28]. It had been also suggested that MVP is virus-induced and has the ability to upregulate type-I interferon production, activate the immune response, and reduce the viral replication, which also supports the role of the vaults in the inflammatory process [25]. MVP also suppresses NF-κB signaling in macrophages [13]. In addition, MVP plays a pivotal role in pro-inflammatory responses. It was found that MVP was essential for the induction of IL-6 and IL-8 [29]. Cells that lack MVP have impaired IL-6 and IL-8 expression, including MVP-deficient human peripheral blood mononuclear cells (PBMCs), human lung epithelial cells, and THP-1 monocytes, as well as murine splenocytes, peritoneal macrophages, and PBMCs from MVP-knockout (MVP−/−) mice [29]. Furthermore, vtRNAs have been suggested to participate in viral replication through inhibition of PKR and as a result of virus replication [30]. In addition, it has been supported that vtRNA1-1 promotes viral establishment and inhibits apoptotic pathways [27]. In our study, MVP levels were found elevated in patients with inflammation as compared to healthy individuals. In addition, the levels of MVP were higher in patients with inflammation of infectious etiology as compared to those with inflammation of non-infectious etiology. It is known that MVP is involved in TLR/IL-1R and TNF-α pathways that that triggers the production of pro-inflammatory cytokines and the onset of inflammation [13]. These pathways overlap when considering the points of activated kinases and transcription factors, as well as the induced expression of inflammatory cytokines [14]. Therefore, increased MVP levels could be produced in the human body in an attempt to reduce inflammation by inhibiting the NF-κB signal transduction. Our previous study revealed that MVP is elevated in patients with rheumatoid arthritis [20]. Since rheumatoid arthritis is a chronic inflammatory disorder that affects joints, MVP may be elevated in this autoimmune disease due to the existence of inflammation. MVP could be also useful for monitoring infections resolving after antibiotic treatment since it is higher in patients with infections than patients with inflammation of non-infectious origin and promptly returns to baseline after the removal of the inflammatory stimulus.

It has also been supported that vaults have a role in cancer and especially in resistance to chemotherapy. Notably, MVP was originally named lung resistance-related protein (LRP) due to its frequent overexpression in multidrug-resistant cancer cells [15,16,31,32,33,34]. Vaults may transport drugs away from their subcellular targets, acting as mediators of drugs’ extrusion from the nucleus and/or their sequestration into exocytotic vesicles [18]. vRNA has also been implicated in this process [35]. The bulk of vRNA in human cells is HVG1, while only small amounts of HVG2 and HVG3 were detected. However, in drug-resistant cancer cell lines, HVG3 was the vRNA found to be mainly associated with the vault particles [11], suggesting that the ratio in which vRNA species are associated with the vaults may be of functional significance. However, in our study, we found no specific correlation of MVP with cancer as it was found elevated also in infections and other types of inflammation. This study has potential limitations. It was not designed to study the response to treatment (e.g., antibiotic treatment in the case of infection). It is also unknown if the elevation in MVP in patients’ serum resulted from necrosis or active secretion of cells. Finally, we were not able to define age- and gender-specific reference values for this new biomarker. MVP shows promise as a novel diagnostic marker, as ROC analysis revealed its diagnostic accuracy is comparable to CRP. Serial sampling analysis in patients demonstrated that MVP levels significantly decreased within the first four days of successful antibiotic treatment for infection, whereas CRP levels showed a less-sensitive decline. This suggests that MVP may offer advantages over CRP in monitoring the progress of antibiotic therapy. Importantly, this study represents the first documentation of MVP as an inflammation marker in the literature.

## 5. Conclusions

In this study, it was found that MVP levels were elevated in patients with inflammation but low in healthy individuals. There was a significant correlation between MVP serum levels and markers of inflammation, such as Neu, CRP, IL-6, and WBCs. Additionally, ROC curves demonstrated that MVP has a diagnostic value similar to the well-established biomarker CRP for diagnosing inflammation. However, gender- and age-specific reference values have to be determined for this novel biomarker prior the detailed evaluation of its diagnostic value. Our study reveals that MVP has the potential to serve as a diagnostic biomarker for both inflammation and infection. Moreover, MVP levels may indicate the efficacy of antibiotic treatment, as they readily return to baseline after the removal of the inflammatory stimulus.

## Figures and Tables

**Table 1:** Demographic and clinical characteristics of study population.

Characteristic	Value
Number of samples	111 (100%)
Healthy individuals	26 (23.4%)
Patients with inflammation	85 (76.6%)
-Infection	36 (32.4%)
-Cancer	25 (22.5%)
-Infection and cancer	3 (2.7%)
-Other	27 (24.3%)
Age (years) ± SD	63 ± 20
Gender	
-Female	33 (29.7%)
-Male	78 (70.3%)
CRP-positive	64.50%

## Data Availability

The original contributions presented in the study are included in the article, further inquiries can be directed to the corresponding author.
